# In Memoriam

**Published:** 1858-12

**Authors:** 


### IN MEMORIAM.

At a meeting of the students of Rush Medical College, on Saturday evening, Jan. 1, 1859, a committee was appointed to express the sentiments of the class relative to the decease of Mr. James J. Kay, one of their number, who reported the following preamble and resolutions, which were unanimously adopted:

Inasmuch as it has pleased Him who disposes all events to remove from our midst by death Mr. James J. Kay, one of our fellow-students, and one who, by his integrity, gentlemanly deportment, kindness of heart, and unremitting diligence in the pursuit of knowledge, had won for himself the respect and esteem of all who knew him, therefore,

*Resolved*, That we sincerely mourn his loss, and deeply sympathize with his afflicted relatives in this hour of their inexpressible grief.

*Resolved*, That we tender to Mr. Atwater and family, and the room-mates of Mr. Kay, our thanks for their prompt, judicious and unceasing attention to the deceased during the whole of his illness.

*Resolved*, That a copy of the foregoing be sent to the parents of the deceased, to his preceptor, and to the daily papers of this city and the Freeport *Journal* and *Bulletin* for publication.

